# The ARIA trial protocol: a randomised controlled trial to assess the clinical, technical, and cost-effectiveness of a cloud-based, *AR*tificially *I*ntelligent image fusion system in comparison to standard treatment to guide endovascular *A*ortic aneurysm repair

**DOI:** 10.1186/s13063-023-07710-5

**Published:** 2024-03-25

**Authors:** James Budge, Tom Carrell, Medeah Yaqub, Hatem Wafa, Matt Waltham, Izabela Pilecka, Joanna Kelly, Caroline Murphy, Stephen Palmer, Yanzhong Wang, Rachel E Clough

**Affiliations:** 1 School of Biomedical Engineering & Imaging Sciences, King’s College London, London, UK; 2 St George’s Vascular Institute, St George’s University, London, UK; 3 Cydar Medical Limited, Cambridge, UK; 4 King’s Clinical Trials Unit, King’s College London, London, UK; 5 Department of Population Health Sciences, King’s College London, London, UK; 6 Centre for Health Economics, University of York, York, UK

**Keywords:** EVAR, FEVAR, BEVAR, Endovascular, Aneurysm, Image fusion, Aortic surgery, AI, Cydar, Computer vision

## Abstract

**Background:**

Endovascular repair of aortic aneurysmal disease is established due to perceived advantages in patient survival, reduced postoperative complications, and shorter hospital lengths of stay. High spatial and contrast resolution 3D CT angiography images are used to plan the procedures and inform device selection and manufacture, but in standard care, the surgery is performed using image-guidance from 2D X-ray fluoroscopy with injection of nephrotoxic contrast material to visualise the blood vessels. This study aims to assess the benefit to patients, practitioners, and the health service of a novel image fusion medical device (Cydar EV), which allows this high-resolution 3D information to be available to operators at the time of surgery.

**Methods:**

The trial is a multi-centre, open label, two-armed randomised controlled clinical trial of 340 patient, randomised 1:1 to either standard treatment in endovascular aneurysm repair or treatment using Cydar EV, a CE-marked medical device comprising of cloud computing, augmented intelligence, and computer vision. The primary outcome is procedural time, with secondary outcomes of procedural efficiency, technical effectiveness, patient outcomes, and cost-effectiveness. Patients with a clinical diagnosis of AAA or TAAA suitable for endovascular repair and able to provide written informed consent will be invited to participate.

**Discussion:**

This trial is the first randomised controlled trial evaluating advanced image fusion technology in endovascular aortic surgery and is well placed to evaluate the effect of this technology on patient outcomes and cost to the NHS.

**Trial registration:**

ISRCTN13832085. Dec. 3, 2021

## Administrative information

Note: the numbers in curly brackets in this protocol refer to SPIRIT checklist item numbers. The order of the items has been modified to group similar items (see http://www.equator-network.org/reporting-guidelines/spirit-2013-statement-defining-standard-protocol-items-for-clinical-trials/).

**Table:** 

Title {1}	The ARIA trial: a randomised controlled trial to assess the clinical, technical, and cost-effectiveness of a cloud-based, *AR*tificially *I*ntelligent image fusion system in comparison to standard treatment to guide endovascular *A*ortic aneurysm repair
Trial registration {2a and 2b}.	The trial has been registered with the ISRCTN, registration number ISRCTN13832085. Registered 3^rd^ December 2021. https://www.isrctn.com/ISRCTN13832085
Protocol version {3}	Protocol Version 1.3, 24.05.2023
Funding {4}	This project is funded by the National Institute for Health and Care Research (NIHR) under its Invention for Innovation (i4i) Programme (Grant Reference Number NIHR201004).
Author details {5a}	Protocol Authors and affiliations: James Budge^1,2^, Tom Carrell^3^, Medeah Yaqub^4^, Hatem Wafa^5^, Matt Waltham^3^, Izabela Pilecka^4^, Joanna Kelly^4^, Caroline Murphy^4^, Stephen Palmer^6^, Yanzhong Wang^5^, Rachel E Clough^1^ 1 School of Biomedical Engineering and Imaging Sciences, King’s College London 2 St George’s Vascular Institute, St George’s University, London. 3 Cydar Medical Limited 4 King’s Clinical Trials Unit, King’s College London 5 Department of Population Health Sciences, King's College London 6 Centre for Health Economics, University of York
Name and contact information for the trial sponsor {5b}	The trial is co-sponsored: King’s College London and Cydar Medical Limited
Role of sponsor {5c}	The study is funded via the above NIHR i4i grant. The study is co-sponsored by King’s College London (KCL) and Cydar Medical Limited. Both co-sponsors were involved in the study design. All trial data collection bar storage of post operative CTs will be performed by KCL trials unit. Post operative CTs will be held by Cydar Medical Limted for analysis by the imaging core lab. Data analysis, interpretation and publication preparation will be the reasonability of KCL with review by Cydar Medical Limited as part of co-authorship of key publications. Health economic analysis will be performed under the supervision of author SP at the University of York.

## Introduction

### Background and rationale {6a}

**Fig. 1:**
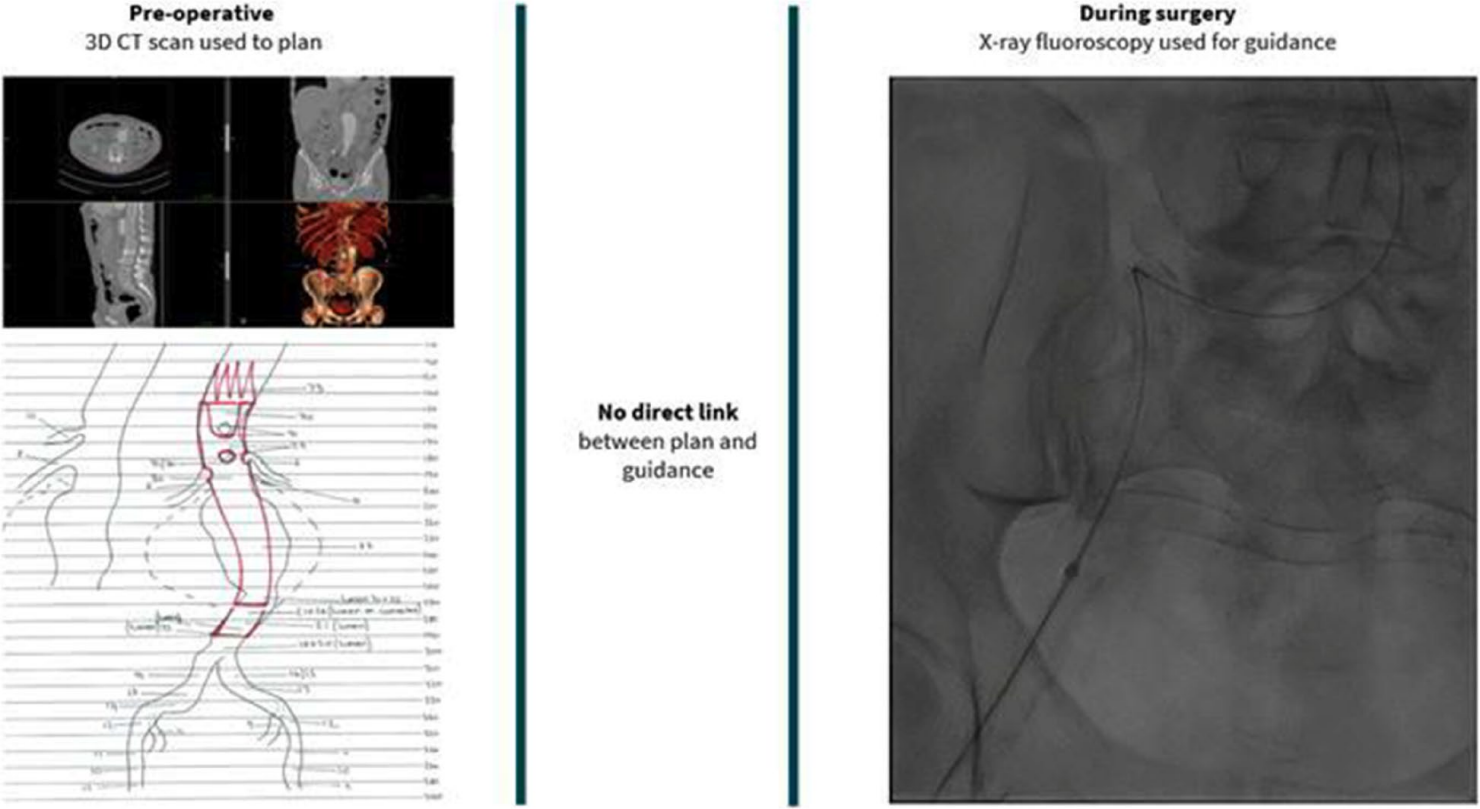
Graphic showing the 3D information the surgeon has preoperatively, and the intra-operative 2D fluoroscopy in current standard of care endovascular surgery

Endovascular aneurysm repair (EVAR) has rapidly replaced open aortic surgery due to perceived advantages in patient survival, reduced postoperative complications, and shorter hospital lengths of stay [1]. Endovascular surgery is planned using 3D reconstructions of pre-operative computed tomography (CT) scans to assess access and determine the optimal type, configuration, and sizing of the implantable medical device. The surgery itself is ‘image-guided’ using 2D X-ray fluoroscopy and injection of nephrotoxic contrast material to visualise blood vessels (see Fig. 1).

Despite the potential advantages of EVAR over open surgery, there are significant concerns related to the variability in planning and sizing, high doses of ionising radiation and nephrotoxic contrast material, imprecise visualisation and device positioning, unpredictability of individual patient outcomes, and inconsistent outcomes between hospitals and regions leading to controversy over cost-effectiveness [2, 3]. Device positioning error can require secondary interventions and cause serious and even fatal complications [4].

**Fig. 2:**
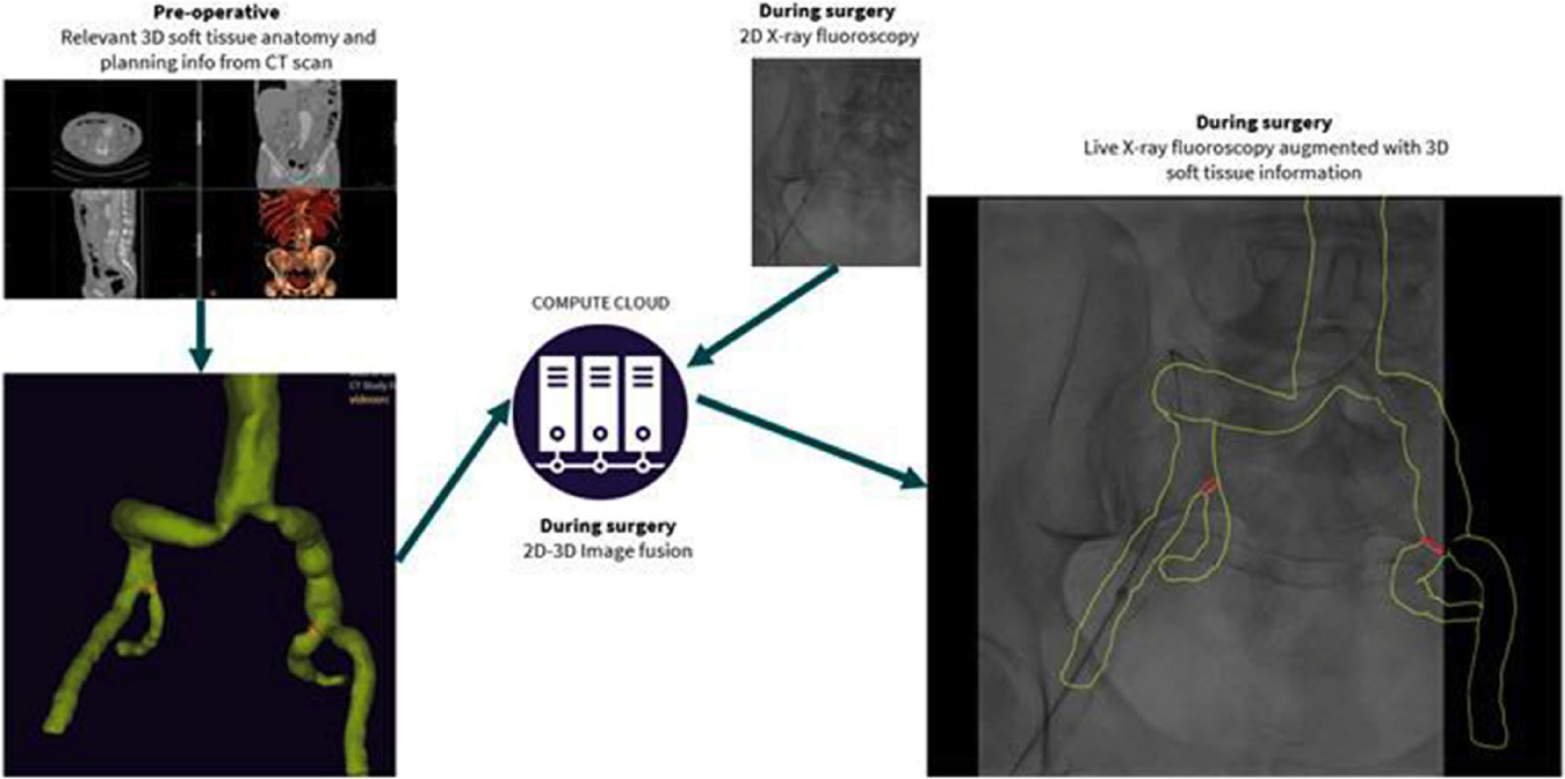
Overview of steps in Cydar EV image fusion; pre-procedural 3D images are fused with intra-operative 2D fluoroscopy automatically and in real-time to produce the combined image shown during the surgery

Previous solutions to improve visualisation during EVAR have included manually aligned, operating table-tracked 3D-2D image overlay. Cydar-EV image fusion is a CE-marked medical device, which instead of a table-tracked overlay uses computer vision to fuse pre-procedural 3D images with intra-operative 2D fluoroscopy automatically and in real-time (see Fig. 2). The key advantage of this type of image fusion is that it gives the surgeon real-time fully integrated 3D visualisation throughout the EVAR procedure with much greater spatial accuracy than achieved by previous technology [5]. The computer vision is a form of artificial intelligence using NHS Digital-approved, GDPR-compliant high-performance cloud computing. Cydar-EV uses only existing patient data (i.e. no new imaging) and is designed not to change clinical workflows. There is no requirement for user interaction, no additional ionising radiation or iodinated contrast. It is agnostic to existing X-ray imaging equipment and can be used on fixed or mobile X-ray systems.

Approximately 5000 EVAR procedures are performed each year in the UK, with an average cost of £19k [6, 7]. EVAR is under an existing NHS care pathway and reduces mortality from 4.7 to 1.7% compared to open surgery, with faster return to normal activities on discharge [1].

Establishing computer vision-powered image fusion as the standard of care in endovascular surgery could directly benefit patients and health and care services by:

1. Reducing procedure times compared to standard procedures and thus improve efficiency of resource use in the NHS
2. Reducing patient exposure to anaesthesia and ionising radiation, lowering surgical site infection, and reducing adverse events
3. Improving procedural success with more precise device positioning compared to current practice
4. Reducing x-ray exposure to patients and staff and reducing the use of nephrotoxic contrast agent, improving renal function
5. Reducing capital expenditure—Cydar-EV can be easily implemented without the need for linked capital expenditure on new fixed imaging or hybrid operating room (cost to NHS ~£2–5m)

A multi-centre observational study (109 patients) examining safety, performance, usability, and efficacy of Cydar-EV was performed 2014–2015. These data were used in the successful application for CE marking. The primary outcomes were as follows:

1. Robustness: 2802 images were analysed, yielding a positive predictive value of 1, with a lower 95% CI of 0.998.
2. Accuracy: tested against the gold-standard data (Tomazevic 2002), the root-mean-square-error was 0.21 mm (max 0.62 mm) [8].
3. Speed: the mean time taken to return and display an updated 3D overlay in response to patient/table/X-ray set movement was 8.395 s (7.232 s excluding network latency); this has since been significantly reduced to < 4 s.
4. Usability: external usability testing in accordance with IEC 62366 validated the display of the 3D overlay information.

Patient benefit was observed by a significant reduction in the amount of X-rays used, with a mean reduction in X-ray fluoroscopy screening time of 35% (*p* = 0.013), a 41% reduction in the amount of iodinated contrast used (*p* = 0.008), and a nearly 1 h reduction in mean operating time (17%, *p* = 0.06) [9]. A further prospective observational cohort study of 119 patients was conducted at Duke University Medical Centre. This reported a mean reduction in procedure time of 17% (*p* = 0.04), with Cydar-EV and a reduction in the number and duration of unexpectedly very long operations [10]. There was also significantly better renal function after the procedure and at 30 days, an indirect metric of the effect of less nephrotoxic contrast agent, and better device positioning. There are no known additional risks of using Cydar EV in comparison to standard treatment. Interruption of the Internet connection during the procedure is possible but rare (< 0.001%).

Demonstrating Cydar-EV improves the outcomes of endovascular surgery at a lower cost for the NHS would be a key demonstration of the potential of digital technology (cloud-computing, big-data, AI) to improve precision and consistency of outcomes for image-guided surgery. It would establish a new concept of data-guided surgery to deliver intelligent planning and outcome analysis, aggregating and learning from existing data to improve the precision, consistency, and transparency of patient outcomes for stakeholders across the NHS: patients, commissioners, hospitals, and clinical teams.

### Objectives {7}

The overarching aim of this trial is to evaluate the clinical, technical, and cost-effectiveness of a novel type of medical device comprised real-time cloud computing, AI, and computer vision (Cydar EV) compared to standard treatment in endovascular aortic aneurysm repair.

#### Primary objective

The primary objective is to assess the effect of Cydar EV on procedure time in comparison to standard treatment in endovascular aortic aneurysm repair.

#### Secondary objectives

The secondary objectives of this study are to evaluate the following:

1. Procedural efficiency, as assessed by:
  a Anaesthetic duration
  b X-ray dose per procedure
  c Contrast dose per procedure
  d Consumable use per procedure
2. Technical effectiveness, as assessed by proximal and distal seal zones at least 10 mm and no evidence of endoleak
3. Patient outcomes, as assessed by:
  a Length of ICU admission
  b Length of HDU admission
  c Post-operative length of hospital stay
  d 30-day mortality
  e Re-intervention—primary hospital visit/further admission (HRG/procedure code)
  f Adverse events (category, LoS, HDU, ICU, general ward)
  g Quality of life (EQ5D)
4. Cost-effectiveness, as assessed by:
  a Total resource use and costs
  b Quality-adjusted life years (QALYs)
  c Incremental cost per QALY

### Trial design {8}

**Fig. 3:**
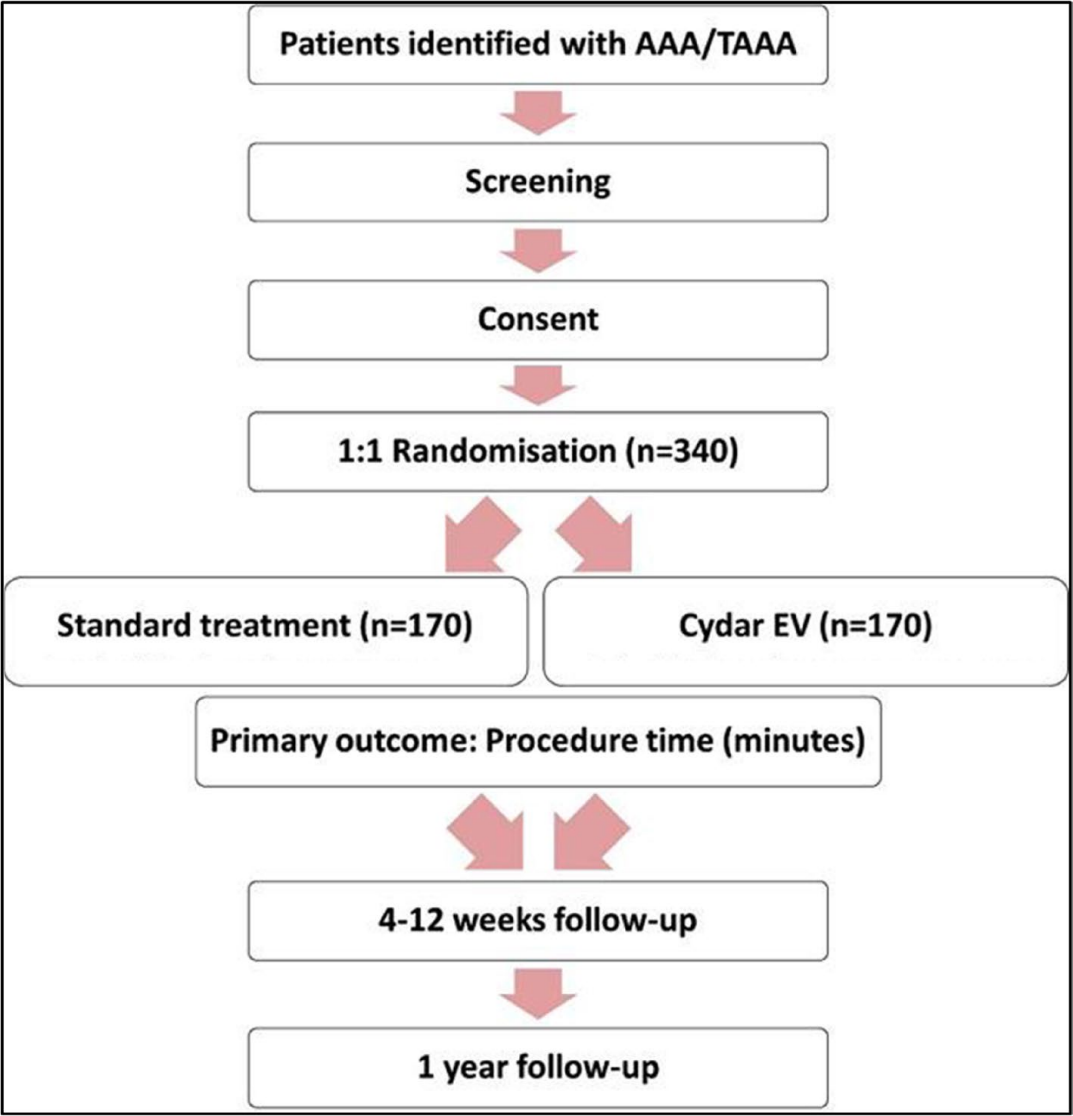
Flow diagram of ARIA trial design

The ARIA trial is a multi-centre, open label, two-armed, parallel groups randomised controlled clinical trial that assigns patients with a clinical diagnosis of abdominal aortic aneurysm and/or thoraco-abdominal aneurysm suitable and fit for endovascular treatment, to either repair using standard treatment or treatment using Cydar-EV. The trial will randomise at a 1:1 ratio and is powered to assess superiority of the intervention. The trial flow diagram is shown in Fig. 3.

## Methods: participants, interventions, and outcomes

### Study setting {9}

The trial will be conducted in 10 centres in the UK over 36 months. Three hundred forty patients will be recruited.

Asymptomatic patients will be identified for inclusion at the time of their clinic appointment, while symptomatic or rupture patients will be identified for inclusion at the time of presentation. Patients that present on an urgent or emergency basis will be required to provide written informed consent, after either reading the patient information leaflet or it being read to them by an individual independent of the trial team and the patient’s family.

The expected recruiting sites are as follows:

1. Leeds Teaching Hospitals NHS Trust
2. Manchester University NHS Foundation Trust
3. Liverpool University Hospitals NHS Trust
4. University Hospital Derby
5. North Bristol NHS Foundation Trust
6. Frimley Health NHS Foundation Trust
7. Imperial Healthcare NHS Trust, London
8. Guy’s and St Thomas’ NHS Foundation Trust, London
9. University Hospital Southampton
10. Brighton and Sussex University Hospitals

### Eligibility criteria {10}

Below are the inclusion and exclusion criteria of the ARIA trial.

Inclusion criteria:

1. Clinical diagnosis of AAA or TAAA suitable for endovascular treatment, as determined by CT imaging and a local treating team multidisciplinary review
2. Patient is confirmed fit for endovascular repair as determined by the operating team
3. CT imaging must be in accordance with Cydar EV: Instructions for Use
4. Written informed consent (patients lacking capacity or unable to speak English will not be enrolled)
5. Age 18 years and above at the time of consent

Exclusion criteria:

1. Patients unable to provide written informed consent

### Informed consent {26a}

Written informed consent will be obtained by the principal investigator or designee at each site, following explanation of the trial procedures. Discussions about trial participation may take place during an in-person consultation or remotely, i.e. during a telephone or video consultation. The participant information sheet can be sent by post or email ahead of the in-person or remote consultation. Full consent will be given in writing, and the signed original consent forms will be retained on site. Randomisation will only take place once the completed consent form has been received and countersigned.

### Additional consent provisions for collection and use of participant data and biological specimens {26b}

No additional consent will be undertaken as part of this trial.

## Interventions

### Explanation for the choice of comparators {6b}

The control comparator used in the ARIA trial will be endovascular aortic aneurysm repair using X-ray fluoroscopy imaging, which represents the reference standard in England.

### Intervention description {11a}

Patients in the intervention arm of the ARIA trial will undergo endovascular aneurysm repair guided by Cydar-EV. Cydar EV provides tools to:

- Import and visualise CT data
- Segment and annotate vascular anatomy from CT data
- Place and edit virtual guidewires and measure lengths on them
- Make measurements of anatomical structures on planar sections of the CT data
- Produce an operative plan from measurements and segmentation of preoperative vessel anatomy
- Overlay planning information such as preoperative vessel anatomy onto live fluoroscopic images, aligned based on the position of anatomical features present in both
- Non-rigidly transform the visualisation of anatomy when intra-operative vessel deformation is observed
- Post-operatively review data relating to procedures where the system was used

#### Intervention training

Training on the Cydar-EV product will be as per the Cydar CE marking and Quality Assurance procedures.

#### Intervention delivery

Procedures will be performed under local, regional or general anaesthesia (likely ratio: 1:1:8). Procedures can be undertaken using either a mobile C-arm in a surgical operating theatre, a dedicated fixed fluoroscopy set, or in a hybrid operating room. Patients may go to the ward, HDU, or ICU following the procedure, according to local protocol. Routine pre-operative CT aortic imaging will be used to determine general suitability for endovascular repair, including assessment of landing zones for fixation and sealing, and procedure type and device selection.

### Criteria for discontinuing or modifying allocated interventions {11b}

Cross-over to Cydar EV from standard care will only be permitted in the context of a procedure duration greater than 8 h or where the patient is in extremis and the surgeon believes that using the Cydar technology may be beneficial to complete the procedure. In these circumstances, the Cydar equipment may be used at the discretion of the operating surgeon, and this information must be captured in the reporting system.

### Strategies to improve adherence to interventions {11c}

Reasons for non-compliance could include Cydar EV device failure, Internet failure, surgeon error, failure to communicate correct randomisation allocation to the surgeon, cross-over, and failure to upload images to Cydar EV, or a non-Cydar-trained surgeon performs the procedure. The patient could impact compliance if they express a wish to withdraw between randomisation and surgical procedure or in the event of death.

### Relevant concomitant care permitted or prohibited during the trial {11d}

There is no restriction on concomitant care during the trial.

### Provisions for post-trial care {30}

Post-trial care will follow routine NHS practice in each centre. In centres where ultrasound imaging is used as the 4–12-week follow-up and/or at 1 year, these patients will be required to undergo one additional CT angiography. This deviation from standard care has been noted in the application for ethical approval for the study.

### Outcomes {12}

#### Primary outcome

Primary efficacy parameter of the study is procedure duration, measured as the time between insertion of the first wire (after percutaneous access achieved, if applicable) at the beginning of the endovascular procedure to the last frame of the completion angiogram. This will be recorded (in minutes) at the time of the procedure by the local research team.

#### Secondary outcomes

1. Procedural efficiency:
  1. Anaesthetic duration—the time between the beginning of induction and the end of emergence. This will be documented at the time of the procedure by the local research team in minutes.
  2. X-ray dose per procedure—fluoroscopy time (FT) (seconds), dose area product (DAP) (Gy.cm2) and cumulative air kerma (CAK) (mGy) should be recorded and documented at the time of the procedure by the local research team. The imaging system used should also be recorded.
  3. Contrast dose per procedure—the volume (ml) and concentration (mgI/ml) of the iodinated contrast material used should be recorded by the local research team at the time of the procedure in minutes.
  4. Consumable use in the operating theatre for endovascular aortic aneurysm repair—name of device, unit and quantity used, blood products used; details to be completed by nurse in the operating theatre or research nurse at the time of the procedure.
2. Technical success:
  1. Proximal and distal seal zone at least 10mm and no evidence of endoleak. This will be documented by the imaging CoreLab team on review of the CT images acquired post-operatively and at 4–12 weeks and at 52 weeks.
3. Patient outcomes:
  1. Length of ICU/HDU admission—date and time from admission to date and time of discharge from ICU/HDU; documented by the local research team during the time of admission; ICU and HDU admissions should be documented separately
  2. Postoperative length of hospital stay—date of procedure to date of discharge from hospital (nights); documented by the local research team during the time of admission.
  3. 30-day mortality—death of the participant within 30 days of the primary procedure; documented by the local research team; to include date of death (dd/mm/yy) and cause.
  4. Re-intervention—any procedure open surgical or endovascular undertaken within 1 year of the primary endovascular aortic aneurysm repair procedure (binary outcome). The type, timing, and number of procedures should also be recorded by the local research team.
  5. Adverse events—hospitalisation for any reason within one year of the primary endovascular aortic aneurysm repair; the type of event should be documented and classified as one of the following: musculoskeletal, urological, neurological, ophthalmological, cardiovascular, gastro-intestinal, hepato-pancreato-biliary, dermatological, or other by the local research team, with information captured to understand if linked to re-intervention. For each hospitalisation, the following should also be captured:
    i. Day case, elective, non-elective
    ii. Length of hospital stay—date of admission to date of discharge (nights)
    iii. Length of ICU/HDU admission (if applicable)—date and time from admission to date and time of discharge from ICU/HDU
  6. Quality of life—differences in quality of life between intervention and the comparator group and changes in quality of life post-surgery will be measured using data from the patient-completed EQ5D-3L [11] instrument. EQ-5D-3L is a validated measure of health-related quality of life, consisting of a five-dimension health status classification system and a separate visual analogue scale. EQ-5D-3L data will be obtained through face-to-face or telephone interview with the participant at baseline, pre-discharge, 4–12 weeks, and at 12 months follow-up.
4. Cost-effectiveness, as assessed by:
  1. Total resource use and costs
  2. Quality-adjusted life years (QALYs)—quality of life will be measured by the EQ-5D-3L instrument as described above. In order to be used in the calculation of quality-adjusted life years (QALYs), the EQ-5D-3L dimension scores will be converted to utilities using the relevant value set for England. Quality-adjusted life years (QALYs) gained in both groups, over the time horizon of the trial, will be calculated using the area under the curve method.
  3. Incremental cost per QALY

### Participant timeline {13}

**Fig. 4:**
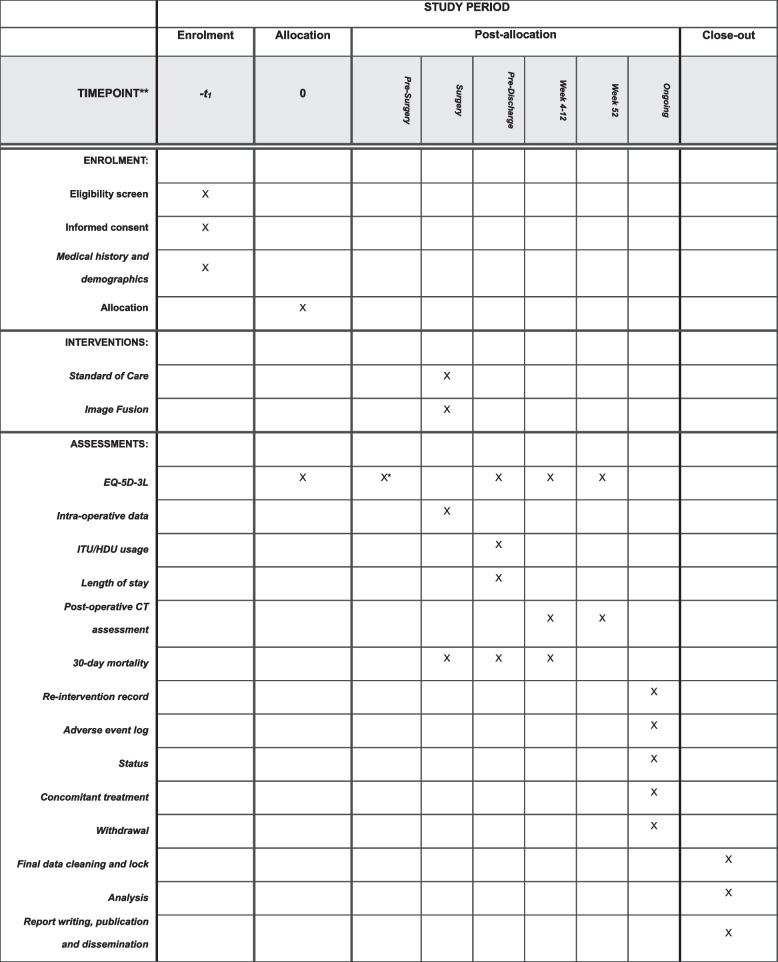
Schedule of enrolment, interventions, and assessments of patients in the ARIA trial

Figure 4 lays out the trial participant time line of enrolment, intervention, and assessment.

### Sample size {14}

There is no known minimum clinically important difference (MCID), and part of the aim of the study is to better characterise the clinical benefit to patients. The study is therefore powered on the basis of a minimum economically meaningful difference. Previous work at Duke [9] reported data on the primary outcome, procedure time, and found a mean difference of 22.5 min (17%) for patients with an abdominal aortic aneurysm treated with Cydar-EV 109.6 (34.2) and standard 2D fluoroscopy imaging 132.1 (69.2) minutes. This is a meaningful difference in the NHS context as this time reduction per case would allow four rather than three EVAR procedures to be performed per day, which is a productivity increase of 33% at the same capacity. The SD for procedure time increases with the mean and so we have assumed a *t*-test for ratio of means 1.2 (fold change), assuming a lognormal distribution for the calculations. Therefore, a sample size of 153 patients per arm with a 1:1 allocation ratio (2 × 153 = 306) would give us 90% power at the 2-sided 5% significance level to detect this difference (PASS 15 Power Analysis and Sample Size Software (2017)). Since our primary outcome measure requires the procedure to be completed, we need to inflate the sample size for possible: (i) loss post randomisation, pre-procedure (est. 7.5%), and (ii) on-table death and cross-overs (where surgeons may use the intervention in a control arm patient and additional assistance is required to complete the operation) (est. 2.5%). These inflate the sample size to 170 per arm. The final randomisation target is therefore 2 × 170 = 340. The Duke data also showed using Cydar-EV in TAAA showed larger reductions in operating time than for AAA. We have powered on the more conservative difference since the relative proportions of AAA/TAAA patients anticipated in our proposed trial is unknown.

### Recruitment {15}

Evidence-based site selection was used to confirm the eligibility of each centre to participate in the trial using volumes of endovascular repair of infra-renal and thoraco-abdominal aortic aneurysms listed on the National Vascular Registry as well as a record of satisfactory patient outcomes and strong clinical engagement. During the trial, the team will maintain regular contact with the sites, undertake regular site visits, and ensure there are adequate numbers of randomisers at sites and that Cydar EV is installed in as many rooms as required. These will be supplemented by in person local principal investigator and research nurse meetings where site teams can hear the experience of other sites and problems and tips and tricks to ensure strong participant recruitment can be shared.

## Assignment of interventions: allocation

### Sequence generation {16a}

Once baseline assessments are complete, participants will be randomised in a 1:1 ratio using the method of minimisation. Randomisation is at the patient level and is performed using a web-based bespoke randomisation system set up by the King’s Clinical Trials Unit (KCTU) at King’s College London. Randomisation is minimised by the following factors:

- Surgeon
- Procedure urgency: emergency or elective
- Procedure type: simple (repair of infra-renal aneurysm ± internal iliac embolisation) or complex (all other types of AAA and TAAA repair, to include branched and fenestrated devices)

The procedure is as follows: on receipt of the baseline questionnaire, the trial coordinator electronically submits details of each participant to the CTU. This includes participant ID number, site, initials, and date of birth. The system immediately notifies the relevant study nurse and records the randomisation outcome. The trial coordinator does not receive the randomisation outcome.

### Concealment mechanism {16b}

Minimisation will incorporate a random component to assure allocation concealment.

### Implementation {16c}

Patients will be enrolled in the ARIA trial by the local trial team at each of the participating sites as per the described consenting procedure. The allocation sequence generation will be implemented via the Kings College Trial Unit (KCTU) web-based randomisation system. The randomisation of participants will be performed post-consent, after checking their eligibility. The signed consent form will be made available for the operating team to review, along with the randomisation result. Participants will be randomised to Cydar-EV image fusion for guidance or standard imaging techniques in a ratio of 1:1 post-consent and confirmation of eligibility.

### Randomisation procedure

Study site staff delegated to undertake the randomisation procedure will generate a unique patient identification number (PIN) using the Elsevier MACRO EDC system and randomise the patient using the KCTU web-based randomisation system.

## Assignment of interventions: blinding

### Who will be blinded {17a}

Due to the nature of the intervention, it is not possible to blind all members of the trial team. Table 1 lays out the blinding status of the research team in this study.

**Table 1:** Blinding status of research team

**Individual blinding status**	**Blinded**	**Unblinded**
Chief investigator	**X**	
Principal investigators at site		**X**
Trial manager/monitor		**X**
Senior statistician	**X**	
Junior statistician		**X**
Independent image reader	**X**	
Cydar project manager		**X**
Trial participants		**X**
Outcome assessors/research nurses		**X**
Treating clinicians		**X**
Trial steering committee (TSC)	**X**	
Data monitoring committee		**X**

### Procedure for unblinding if needed {17b}

Emergency unblinding is not required in this study.

## Data collection and management

### Plans for assessment and collection of outcomes {18a}

#### Source data worksheets

Sites will be provided with source data worksheets containing the relevant data required to be transcribed to the MACRO EDC system and the randomisation system. Training will be provided by the ARIA trial manager.

#### CT aorta image reading

CT imaging data will be uploaded to the ARIA trial image analysis virtual CoreLab, which is a cloud-based system. Images will be read in a blinded manner by two readers, with more than 5 years of experience of aortic image analysis and with experience of aortic endovascular surgical planning. They will securely log into the cloud-based Cydar vault where the CT image data will be housed and analyse the pre- and post-operative CTs to determine technical success. Data from this analysis will be entered onto a part of the MACRO EDC system inaccessible to sites. Twenty image data sets will be used to assess the inter- and intra- observer repeatability coefficients for the variables in the CT read protocol.

#### Plans to promote participant retention and complete follow-up {18b}

Participants will be seen in routine NHS follow-up clinics. If visits have not been scheduled by the end of the week 4–12 and week 52 visit windows, the study site staff will contact the participants by telephone to collect the EQ-5D (telephone version) and follow-up data, and attempts will continue to schedule a follow-up visit. Data will be collected and entered, even if follow-up clinic assessments are outside the optimal visit windows and the date noted.

### Data management {19}

#### Data entry

Authorised staff at sites will transcribe baseline and follow-up data from the source data worksheets. A full audit trail of data entry and any subsequent changes to entered data will be automatically date and time stamped, alongside information about the user making the entry/changes within the system.

#### Security (EDC)

Systems access will be strictly restricted through user-specific passwords to the authorised research team members. Participant initials and partial date of birth (mm/yyyy) will be entered into the systems. Hospital number, email address, participant names and addresses, and full postcodes will not be entered into the EDC system. Trial sites will maintain a master patient log linking participant identifiers to study numbers. No data will be entered unless a participant has signed a consent form to participate in the trial.

#### Data quality processes

At the database design stage, validations will be programmed into the systems to minimise data entry errors by querying the data entered in real time with sites. The CI team will undertake appropriate reviews of the entered data, in consultation with the project analyst, where appropriate for the purpose of data cleaning and will request amendments to the MACRO EDC system data as required. No data will be amended independently of the study site responsible for entering the data. No data can be amended in the randomisation system; however, CI or delegate (e.g. trial manager) may request King’s Clinical Trials Unit to add notes against individual participant entries to clarify data entry errors. Any errors should be reported by site staff to the trial manager as soon as possible once they are detected. The trial manager will onward report errors to KCTU and retain records in the TMF. Site monitoring visits will be conducted by the trial manager.

### Confidentiality {27}

When consent forms are signed, a copy will be provided to the patient, a copy will be filed in the medical records, and the original will be retained in the Investigator Site File. Participant initials and date of birth will be entered into the study database, but no more identifying information will be collected outside the recruiting study site. Within site, an Investigator Site File will be maintained by the site PI. Participants will be fully identifiable within these files.

### Plans for collection, laboratory evaluation, and storage of biological specimens for genetic or molecular analysis in this trial/future use {33}

There are no biological specimens that will be taken as part of this trial.

## Statistical methods

### Statistical methods for primary and secondary outcomes {20a}

The analyses will be carried out according to the statistical analysis plan written before any outcome data are inspected. A CONSORT diagram will describe the patient flow and exclusions. Baseline demographic and clinical data will be summarised by randomisation trial arm.

#### Statistical methods for primary outcome

As the primary outcome is procedure duration and we envisage 7.5% loss of patients between randomisation and procedure, the primary analysis will be a per-protocol (PP) analysis based on procedure time. The primary analysis will be conducted after completion of first follow-up (at 4–12 weeks) which will include procedure time as well as the secondary outcome data available at this time. Sensitivity analysis with multiple imputation for missing data will also be conducted alongside the per-protocol analysis. No significance tests will be performed for baseline comparison. The primary outcome measure is likely to have a skewed distribution and therefore if necessary and possible the data will be normalised using an appropriate transformation. The data will then be analysed using linear regression techniques with stratification (minimisation) factors included as covariates. If a suitable transformation cannot be found, the data will be analysed using quantile regression to allow us to include the addition of the stratification factors as covariates.

#### Statistical methods for secondary outcomes

A similar analysis will be undertaken for the secondary outcomes including quality of life scores. Binary outcomes will be compared between arms using logistic regression adjusting for stratification factors. Outcomes will be reported as adjusted differences in means (or median) or odds ratios for continuous and binary data respectively. All tests will be two sided and will be assessed at the 5% significance level. Safety outcomes will be reported as patient proportions and rates within and between arms with 95% confidence intervals using exact methods where appropriate.

### Interim analyses {21b}

There will be no planned formal interim analyses of the primary and secondary outcomes. However, we will conduct further analyses of secondary outcomes at the completion of the 52-week follow-up for all the patients.

### Methods for additional analyses (e.g. subgroup analyses) {20b}

We will use data collected during this study for further analyses investigating the following topics:

i. Image analysis—a CoreLab will perform imaging analysis of technical outcomes and anatomy as seen on the 2 postoperative CTs.
ii. Health economic analysis will be performed to examine the cost-effectiveness of the Cydar EV system. This will include an analysis of the systems efficiency that the Cydar EV system may allow.
iii. Quality of life between the two groups will be assessed using the area under the curve method.

### Methods in analysis to handle protocol non-adherence and any statistical methods to handle missing data {20c}

Compliance with intervention will be recorded in the source data worksheets and transcribed to the EDC system. Reasons for non-compliance would include device failure, surgeon error, or failure to communicate correct randomisation allocation to the surgeon. The patient could only impact compliance if they express a wish to withdraw between randomisation and surgical procedure. Missingness will be reported and reasons for missingness explored. Although a low percentage of missing data is anticipated, a sensitivity analysis of the primary outcome will be undertaken in order to assess the impact of the exclusion of participants with missing intraoperative data in the primary analysis. In this view of the sample size, a modelling approach will be taken rather than multiple imputation.

### Plans to give access to the full protocol, participant-level data, and statistical code {31c}

The investigator(s) will permit trial-related monitoring, audits, and REC review by providing the sponsor(s) and REC direct access to source data and other documents (e.g. patients’ case sheets, blood results, imaging reports, trial protocol, statistical code, and etc.).

## Oversight and monitoring

### Composition of the coordinating centre and trial steering committee {5d}

The trial will be coordinated through the KCTU with the CI being supported by the trial management group (TMG) who are made of the following members: Cydar lead investigator, KCTU operations director, KCTU data centre lead, KCTU senior statistician, KCTU junior statistician, KCTU trial manager, trial health economist, Cydar project manager, and the KCL clinical research fellow. The TMG is responsible for the study coordination, data quality, and budget management. The TMG members will meet at least monthly throughout the trial. The CI will chair the TMG. Minutes will be taken by the trial manager and retained in the TMF. The TMG will review recruitment to the study across all study sites and will take appropriate action in the event the study recruitment rate is lower than anticipated.

The TSC is an executive committee, reporting to the funder (NIHR) and the sponsor. Independent members will be independent of both the sponsor organisations and of any recruiting study sites. The terms of reference of the TSC will be agreed at the first meeting, prior to start of recruitment. Meetings will be scheduled approximately 2 weeks after each data monitoring committee (DMC) meeting. Minutes will be taken by the trial manager and retained in the TMF. The trial manager will prepare reports to the TSC. The trial may be prematurely discontinued by the co-sponsors or chief investigator on the recommendation of the trial steering committee.

### Composition of the data monitoring committee, its role and reporting structure {21a}

The data monitoring committee (DMC) will be composed of three independent members: a statistician and two clinicians. The DMC is an advisory committee, reporting to the trial steering committee. They will receive a report of recruitment and serious and non-serious adverse events and a summary of accumulated clinical data from the trial statistician and will meet in person or by telephone. The DMC will meet at least annually during the study, approximately 2 weeks prior to the TSC. Members will be independent of the sponsor organisations and of any recruiting study sites. The DMC will work to the DAMOCLES guidance and a DMC charter will be agreed at the first meeting outlining responsibilities, reporting, meeting frequency, documentation, and other matters. The trial statistician will prepare reports for the DMC.

### Adverse event reporting and harms {22}

Adverse events will be categorised as per the medicines for human use (Clinical Trials) regulations 2004 and amended regulations 2006. Where any adverse event occurs, the relationship with the investigational product will be assessed to determine the relationship and be judged as, definitively, probably, possibly, unlikely, not related, or not assessable. All SAEs, SARs, and SUSARs will be reported immediately (and certainly no later than 24 h) by the investigator to KCTU. The chief investigator will report relevant SAEs to the ethics committee.

### Frequency and plans for auditing trial conduct {23}

Monitoring of this trial will ensure compliance with Good Clinical Practice and will be managed by the trial manager at King’s College London. The investigators will permit trial-related monitoring, audits, REC review, and regulatory inspections by providing the sponsors, regulators, and REC direct access to source data and other documents (e.g. patients’ case sheets, blood results, imaging reports, trial protocol, statistical code, and etc). KCTU will prepare a monitoring plan for approval by the TMG. Recruiting study sites will have a site initiation visit prior to recruitment of the first participant and regular site visits thereafter to verify the data.

### Plans for communicating important protocol amendments to relevant parties (e.g. trial participants, ethical committees) {25}

The trial manager will be responsible for preparing and submitting protocol amendments to the ethics committee and the HRA and circulating updated document versions to recruiting study sites, co-applicants, the TMG, TSC, and DMC, and (where relevant) the funder. Site investigators will be responsible for communicating relevant information to study participants.

### Dissemination plans {31a}

The primary analysis will be conducted after completion of first follow-up (at 4–12 weeks) which will include procedure time as well as the secondary outcome data available at this time and published in a peer reviewed open source medical journal as early as possible. The 52-week secondary outcomes will be published in a further paper when all outcome data collection is complete. Recruiting sites will be informed of the results and will be asked to disseminate the findings to participants. Patient groups will be informed of the results for dissemination among their members. The sharing dataset will be passed to the trial chief investigator by the analyst, and all future data sharing will be managed as per the head contract and associated collaboration agreements.

## Discussion

Not applicable. We have no practical or operational issues to report that involve performing the study.

## Trial status

The current trial protocol version is 1.3 and was published on 24 May 2023. Recruitment began 4 May 2022 and is expected to be completed by 31 December 2023.

## Data Availability

Data will be available for sharing upon request for future scientific research, subject to approval by the co-sponsors.

## Abbreviations

AAA: Abdominal aortic aneurysm
CI: Chief investigator
CT: Computerised tomography
DMC: Data monitoring committee
EDC: Electronic data capture
GP: General practitioner
GCP: Good clinical practice
HDU: High-dependency unit
ICU: Intensive care unit
ITT: Intention to treat
KCTU: King’s Clinical Trials Unit
MHRA: Medicines and Healthcare products Regulatory Agency
PI: Principal investigator
PIS: Participant information sheet
PP: Per protocol
REC: Research ethics committee
RN: Research nurse
TAAA: Thoraco-abdominal aortic aneurysm
TM: Trial manager
TMG: Trial management group
TS: Trial statistician
TSC: Trial steering committee
